# The relationship between cannabis use, depression, anxiety and self esteem among Tunisian young adults living abroad

**DOI:** 10.1192/j.eurpsy.2024.834

**Published:** 2024-08-27

**Authors:** M. Moalla, N. Charfi, H. Ktari, S. Omri, N. Smaoui, I. Gassara, R. Feki, L. Zouari, M. maalej bouali, J. Ben Thabet, M. Maalej

**Affiliations:** ^1^Psychiatry C, Hedi Chaker Hospital; ^2^Psychiatry C, Hedi Chaker Hospital of Sfax, Sfax, Tunisia

## Abstract

**Introduction:**

Cannabis use is very common worldwide. Its consumption could be explained by recreational purposes as it can be motivated by some psychiatric disorders such as depression, anxiety or low self-esteem.

**Objectives:**

This study aims to explore the relationship between cannabis use, depression, anxiety and self-esteem among a population of Tunisian adults living abroad.

**Methods:**

A cross-sectional online survey was carried out using a self-administered questionnaire on young Tunisians people who have completed their secondary studies at the pilot high school of Sfax and currently residing abroad. The survey questionnaire was designed on Google Forms. It included a data collection sheet and psychometric scales “Cannabis Abuse Screening Test” (CAST), “Hospital Anxiety and Depression Scale” (HADS) and”Rosenberg’s self-esteem scale”.

**Results:**

The sample consisted of 35 Tunisian young adults. 17 participants (48.6%) reported a cannabis use behavior. It was done with friends in a festive setting in 88% of cases (N=15). According to the CAST, 17,6% (N=3) of cannabis users were at high risk of cannabis dependence. Anxiety was present in 17,6% (N=3) and depression in 17,6% (N=3) of participants. Self-esteem was low in 23,53% (N=4) of participants.

Cannabis use was not associated with the presence of current emotional disorders such as anxiety and depression (p=0.894 and p=0.933 respectively). It was also not associated with lower self-esteem (p=0.585).

**Conclusions:**

Cannabis use is relatively common among young Tunisian emigrants without evolving towards dependence inseveral cases. This behavior seems to be more influenced by social factors and misrepresentations about cannabis than by psychological disturbance.

**Disclosure of Interest:**

None Declared

